# 7 T and beyond: toward a synergy between fMRI-based presurgical mapping at ultrahigh magnetic fields, AI, and robotic neurosurgery

**DOI:** 10.1186/s41747-024-00472-y

**Published:** 2024-07-01

**Authors:** Mohamed L. Seghier

**Affiliations:** 1 Department of Biomedical Engineering and Biotechnology, Khalifa University of Science and Technology, Abu Dhabi, UAE; 2 Healtcare Engineering Innovation Center (HEIC), Khalifa University of Science and Technology, Abu Dhabi, UAE

**Keywords:** Artificial intelligence, Brain mapping, Echo-planar imaging, Magnetic resonance imaging, Robotic surgical procedures

## Abstract

Presurgical evaluation with functional magnetic resonance imaging (fMRI) can reduce postsurgical morbidity. Here, we discuss presurgical fMRI mapping at ultra-high magnetic fields (UHF), *i.e.*, ≥ 7 T, in the light of the current growing interest in artificial intelligence (AI) and robot-assisted neurosurgery. The potential of submillimetre fMRI mapping can help better appreciate uncertainty on resection margins, though geometric distortions at UHF might lessen the accuracy of fMRI maps. A useful trade-off for UHF fMRI is to collect data with 1-mm isotropic resolution to ensure high sensitivity and subsequently a low risk of false negatives. Scanning at UHF might yield a revival interest in slow event-related fMRI, thereby offering a richer depiction of the dynamics of fMRI responses. The potential applications of AI concern denoising and artefact removal, generation of super-resolution fMRI maps, and accurate fusion or coregistration between anatomical and fMRI maps. The latter can benefit from the use of T1-weighted echo-planar imaging for better visualization of brain activations. Such AI-augmented fMRI maps would provide high-quality input data to robotic surgery systems, thereby improving the accuracy and reliability of robot-assisted neurosurgery. Ultimately, the advancement in fMRI at UHF would promote clinically useful synergies between fMRI, AI, and robotic neurosurgery.

**Relevance statement** This review highlights the potential synergies between fMRI at UHF, AI, and robotic neurosurgery in improving the accuracy and reliability of fMRI-based presurgical mapping.

**Key points**

• Presurgical fMRI mapping at UHF improves spatial resolution and sensitivity.

• Slow event-related designs offer a richer depiction of fMRI responses dynamics.

• AI can support denoising, artefact removal, and generation of super-resolution fMRI maps.

• AI-augmented fMRI maps can provide high-quality input data to robotic surgery systems.

**Graphical Abstract:**

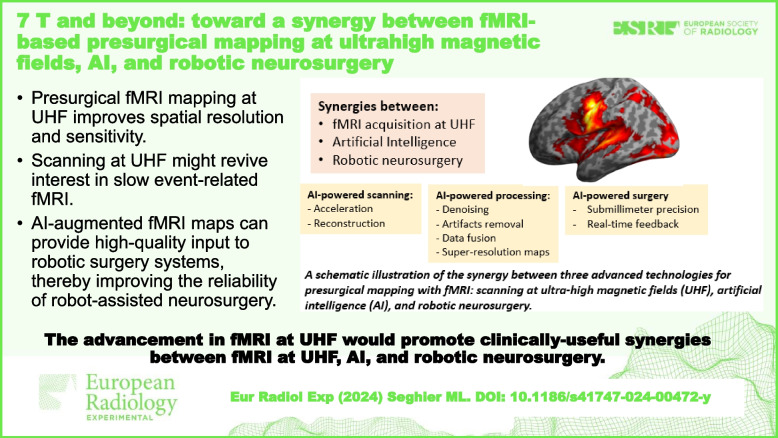

## Introduction

In clinical settings, presurgical mapping of eloquent cortex is usually achieved with invasive procedures. For example, intraoperative brain mapping on awake patients by cortico-cortical evoked potential involves direct cortical stimulation to observe elicitation of an impairment with high frequency stimulation (25 to 50 Hz) or evoked responses in distant or nearby cortical regions with low-frequency stimulation (1 Hz). This invasive procedure can sometimes yield behavioral responses that are difficult to interpret [1, 2].

An alternative approach that is relatively noninvasive is functional magnetic resonance imaging (fMRI). This technique has been shown to provide robust and reliable assessment of brain function with typical clinical MRI scanners at 1.5 or 3 T [3]. Indeed, presurgical mapping with fMRI remains the most widely used application of clinical fMRI [4, 5], enabling patient-specific mapping of eloquent brain regions close to pathologies [6], including brain tumors or epileptic foci, so that vital activated brain regions are spared to minimise the risk of postsurgical impairments. There is compelling evidence that preoperative mapping with fMRI can reduce postsurgical morbidity [7, 8].

fMRI-based presurgical mapping is considered one of the key applications that will largely benefit from scanning patients at ultrahigh magnetic fields (UHF) [9–12]. Although task-based and task-free fMRI is still widely performed at traditional field strengths (1.5 or 3 T), fMRI at UHF (7 T or above) is rapidly gaining in popularity after 7-T MRI scanners were approved for clinical use by the FDA and the European Union, and many studies have already shown that presurgical mapping at UHF is safe [13, 14]. Brain mapping at UHF boosts reliability at the individual patient level thanks to the increase in signal-to-noise ratio (SNR) [15, 16] and the subsequent enhancement in contrast-to-noise ratio [17, 18]. This increase in SNR can translate into (i) higher spatial resolution, (ii) higher temporal resolution, (iii) shorter total acquisition times, (iv) better blood oxygenation level dependent (BOLD) sensitivity to minimise false negatives, and (v) improved BOLD spatial specificity by reducing signals from draining veins. Here, we discuss how UHF can transform current presurgical mapping procedures in the clinical setting, in particular in the current growing interest in AI and robotic surgery.

It is well documented that high spatiotemporal brain mapping at UHF [18] allows better specificity with significantly less partial volume effects compared to fMRI at traditional field strengths [19]. For instance, previous presurgical fMRI studies have demonstrated a significant increase in sensitivity with motor [20] and language tasks [21] as well as during rest [22] at UHF. The superiority of presurgical brain mapping at UHF compared to traditional field strengths is observed in terms of a higher number of suprathreshold voxels or clusters, a larger percent signal change or effect size, a higher statistical *t*-values or *z*-scores, and/or the activation of small or deep structures that are sometimes difficult to depict at traditional field strengths [23–26]. Such gains are a direct consequence of the monotonic increase of BOLD sensitivity with magnetic field strength, yielding increased extent of activated areas at higher spatial definitions. This has significant practical implications for neurosurgery: (i) higher spatial resolution fMRI maps offer the possibility for resections at an excellent precision, (ii) larger activated volumes might presumably translate into more conservative resections, and (iii) high BOLD sensitivity and low false negative rates would translate into reduced risk of postsurgical complications. However, despite such significant benefits at UHF, we argue here that the most beneficial aspect of conducting presurgical mapping at UHF should concern the increase in BOLD sensitivity rather than a push for higher sub-millimetric spatial resolutions, in particular in the light of the strong susceptibility-related distortions at UHF.

## Optimal fMRI paradigms at UHF

All types of fMRI paradigms are applicable at UHF. Task-based fMRI with block, event-related, or mixed designs offer flexibility in mapping brain function according to the function of interest and the patient’s ability to perform the task [27]. The relevant features that define an optimal design are the same as those at traditional magnetic fields, including stimulus type, baseline condition, task, response type, and acquisition duration, given that many of these features can impact on the accuracy of presurgical fMRI, as shown in a systematic review [28]. Moreover, studies have tested and compared a variety of tasks to identify the most reliable tasks and paradigms for presurgical evaluation [29–31]. This has led to the publication of several recommendations and guidelines about the optimal tasks to use at traditional fields [32, 33]. Many of these recommendations are still valid and useful at UHF. However, it is likely that some tasks previously described as less reliable at traditional fields might be useful at UHF due to the increase in BOLD sensitivity. This calls for an update to existing guidelines, which might help expand the repertoire of motor and cognitive tasks that can be used in fMRI-based presurgical evaluation at UHF. For example, in the language domain, reading or semantic tasks are not typically considered as highly reliable tasks for presurgical identification of temporal regions [34–36]. It is likely that such tasks, even when used with passive responses, could still be valuable at UHF for some patients who struggle with tasks that rely on word finding.

Regarding task-free fMRI, mapping at rest is particularly useful for patients who are unable to cooperate or who are scanned under sedation [37, 38], with the advantage of localizing many brain regions and networks from one session/run [39, 40]. Presurgical mapping with resting-state fMRI has been shown to be reliable and concordant with task-based fMRI as well as with intraoperative electrocortical stimulation [40–43]. Recent work reported high reliability (*i.e.*, high between-session consistency and stability) of single-subject resting state networks at UHF [44, 45]. Likewise, resting-state fMRI demonstrated a superior seizure onset-zone lateralizing ability at 7 T compared to 3 T during an epilepsy presurgical evaluation [22]. Despite its potential in presurgical mapping, the application of resting-state fMRI is still facing many methodological challenges with respect to the complexity of data analysis, the lack of standardization of fMRI protocols at rest [46] and the difficulty to assign a specific function of a particular node of a resting-state network [47]. Likewise, there is a lack of awareness among clinicians about the potential of such protocols at rest and the availability of automated data processing methods at the single patient level [48]. Nevertheless, fMRI at rest will likely gain in popularity at UHF, making the mapping of different brain networks at the individual patient level from one session (5−10 min) easily manageable by patients and MRI technicians.

In the same way, naturalistic fMRI paradigms offer an attractive alternative for presurgical fMRI mapping. They can reliably depict eloquent cortex, including paradigms that involve passive viewing of movie clips [49, 50]. Such paradigms are very handy when patients cannot perform tasks or cooperate in the absence of any external instructions. They also help increase scanner tolerability when scanning with noisy sequences at UHF and can decrease boredom and anxiety in the scanner. As patients are not asked to actively respond to (repetitive) stimuli, watching video clips can minimise the occurrence of head motion artefacts. Video clips can be tailored to the specific function or the population of interest. For instance, different video clips can be used in presurgical mapping in elderly patients with tumors or young children with epilepsy. Another benefit of video clips is that they can be translated to any language and are easy to share across different MRI sites. It is expected that movie fMRI-based presurgical mapping will gain in popularity at UHF.

**Fig. 1:**
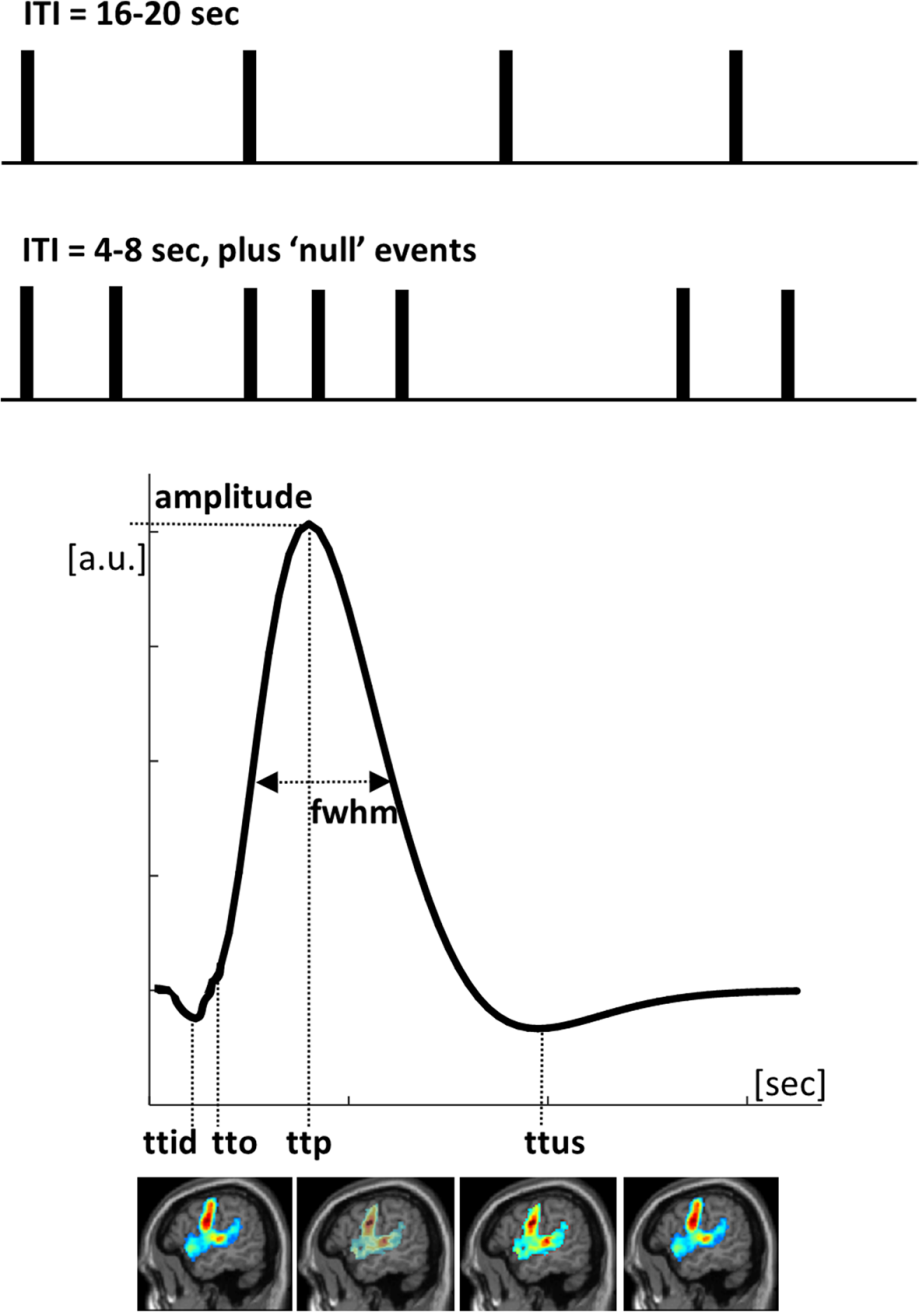
Slow (top) and fast (middle) event-related design. Events or trials are shown with black bars. Deconvolution methods can be used with fast event-related fMRI, assuming linear responses, low inter-trial variability, and uncorrelated noise. When these conditions are not met, slow event-related designs are preferable as any loss in paradigm design efficiency is compensated by the high signal-to-noise ratio at ultrahigh field. Bottom: an example of a typical hemodynamic response function with some useful parameters that can be derived from it: ttid (time to initial dip), tto (time to onset), ttp (time to peak), ttus (time to undershoot), fwhm (fullwidth at half maximum). These parameters can be estimated at each voxel, yielding multiple three-dimensional maps for each task/contrast. *fMRI* Functional magnetic resonance imaging, *ITI* Intertrial interval

Now, we turn to an overlooked paradigm that concerns mapping brain activations with slow event-related designs. Although rapid event-related designs offer higher detection power than other paradigms [51], slow event-related design might transform how presurgical mapping is carried out. Thanks to the increase in SNR at UHF, slow event-related designs, defined as event related paradigms with an interstimulus interval larger than the typical duration of the hemodynamic responses function [52], can identify not only the activated regions but also their estimated hemodynamic delays [53]. With minimal overlap between BOLD responses of successive trials in slow event-related designs, different time parameters and latencies [54–57] that characterise the dynamics of the hemodynamic correlates of neuronal function in patients can be estimated (Fig. 1), which could be useful given the different biological and methodological factors that impact on the modelling of BOLD responses at high spatial resolution [58]. Such time parameters can offer an insight into tumor-induced alterations to the neurovascular coupling [59, 60]. For instance, multiple whole-brain maps can be generated for a given task at the individual patient level, including maps of time-to-onset, time-to-peak, response width, and peak amplitude [61]. This would help explain instances of aberrant fMRI responses in patients [62] and to better appreciate the risk of false negatives and false positives in presurgical fMRI. Perhaps most importantly, the multiple generated maps provide richer information at the voxel level that can be valuable for the development of risk prediction models about post-surgery outcome and recovery.

## The challenge of submillimetre presurgical mapping

**Fig. 2:**
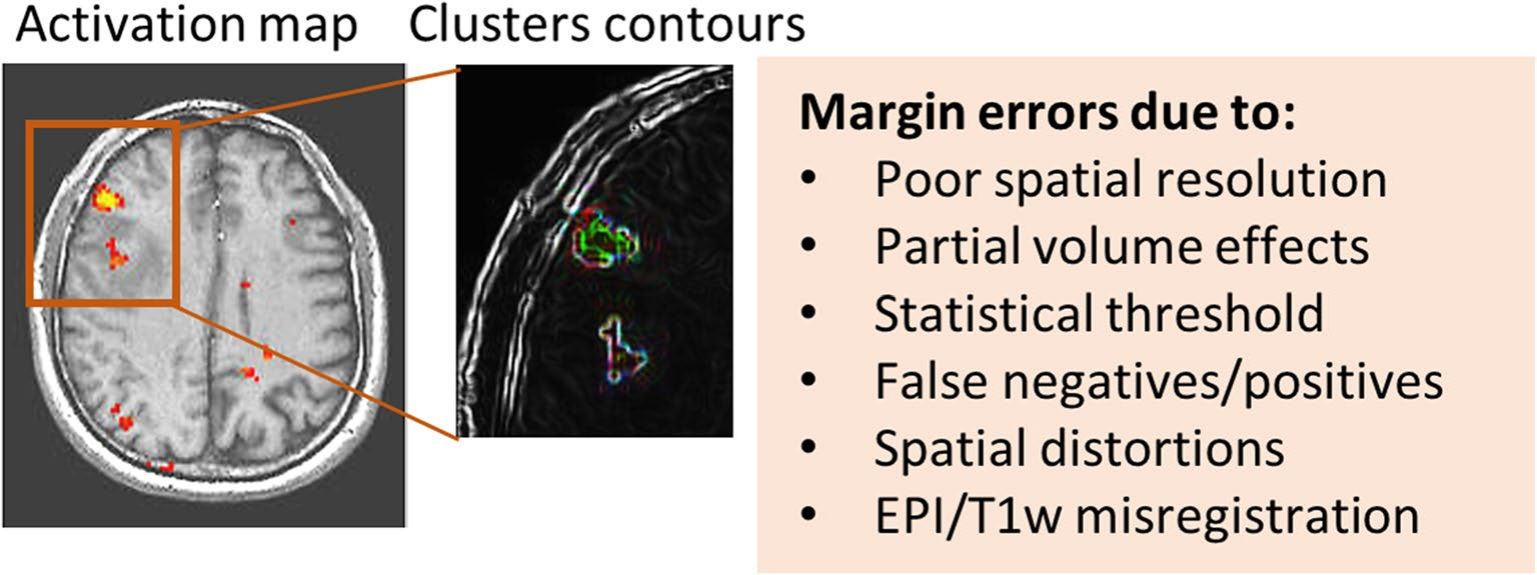
fMRI activations in a patient with a brain tumor (showing one slice dorsal to the main tumor in the right hemisphere). A zoomed view showing the contours of two activated clusters. The location and extent of the clusters are important indicators to be accounted for during the definition of the resection margins. However, location and extent of activations are intrinsically defined by many factors including the original spatial resolution, partial volume effects in particular near tissue borders as well as the heterogenous peri-tumoral zone, the effect of the statistical threshold, the risk of false negatives and positives that can be exacerbated by artefacts such as head motion artefacts, and susceptibility artefacts causing geometric distortions that can lead to a mismatch or misregistration between functional echo-planar imaging (EPI) and anatomical T1-weighted (T1w) images. *fMRI* Functional magnetic resonance imaging

Submillimetre presurgical mapping of brain function is often portrayed as the most beneficial aspect of scanning patients at UHF. This gain in spatial resolution would enable resection of brain tumors or epileptic foci at high spatial precision, thus minimizing the extent of postsurgical damage to neighbouring healthy tissue. For that purpose, the localization of eloquent cortex needs to be highly accurate because a small shift of resection margins or suboptimal surgical access can yield postsurgical impairments (Fig. 2). For instance, it has been shown that resection margins in the millimetre range close to eloquent brain areas may determine whether postoperative deficits are reversible or permanent [63], which underscores the importance of high-resolution brain maps for image-guided surgeries.

However, the precision that would be gained for neurosurgical procedures is inherently bounded by the accuracy of the fMRI-based mapping of the eloquent cortex at UHF. Despite the possibility to scan the brain at submillimetre scale, the presence of susceptibility artefacts can induce geometric distortions that yield tissue displacements in the collected images [64]. This is because brain tissues with differing magnetic susceptibilities can cause gradients in the static magnetic field, which would lead to regional variations in the effective echo time, resulting in artefacts in image signal and BOLD sensitivity [65]. For instance, the widely used echo-planar imaging (EPI) in fMRI is prone to geometric distortions [64, 66], yielding clinically significant displacements of brain activations [67]. Such spatial distortions are notoriously difficult to correct at UHF as they do increase with magnetic field strength, so it is of paramount importance that these distortions are corrected at UHF. Furthermore, such spatial distortions might be particularly problematic when scanning patients at UHF, even when other acquisition schemes such as spiral fMRI is used [68] or multiband EPI [69], as they get worse with large head motion artefacts in patients and they tend to be spatially heterogenous [70] as in the case of severe distortions in brain areas around air cavities or distant from the isocenter of the scanner [71].

Despite the existence of many sophisticated correction methods for geometric distortions [67, 72–74], still displacement errors after correction are above the millimetre level in some brain regions, making any ultra-high-resolution fMRI mapping (*e.g.*, voxel size < 1 mm^3^) not very reliable for surgical applications. For instance, a recent comparative study showed possible displacement of up to 4 mm in raw EPI images, in particular in ventromedial prefrontal regions, that were corrected to the voxel size using different corrections methods [74]. Another study reported spatial distortions up to 5.1 mm in the primary motor cortex in raw EPI images, but these distortions were reduced to less than 1.7 mm after correction [67]. Likewise, correction for susceptibility artefacts based on T1-weighted anatomical images was shown to be highly robust when tested on a submillimetre 7-T fMRI dataset [75]. In addition to susceptibility-induced distortions, alterations to the vasculature of the brain in the presence of tumors could adversely affect the neurovascular coupling [60], especially in peritumoral areas, which might yield to spatially aberrant brain activations. Taken together, these effects would limit the reliability of submillimetre fMRI mapping at UHF, suggesting that going below isotropic 1-mm spatial resolution in fMRI might not necessarily translate into accurate submillimetre resection margins. Advanced correction methods are needed in order to generate artefact-free fMRI maps at UHF, including new methods based on AI.

## AI-based fMRI enhancement and artefacts correction

In this context, one exciting emerging field concerns the application of machine learning techniques, including deep learning, for the correction of artefacts in MRI [76, 77]. AI-based corrections for motion artefacts [78, 79] and susceptibility artefacts in EPI images [80–83] have been shown to be robust and extremely useful. The appealing feature in these techniques is that they do not require prior knowledge on the exact true mapping between brain structures and MRI-based depiction of those structures, as this (nonlinear) mapping is learned in a data-driven way. This is very useful in the context of fMRI as artefacts are extremely complex to fully characterise because they are spatially heterogenous, variable across subjects, sessions, sequences, and scanners, and they also result from an intricate interplay between different interacting sources including geometric distortions, motion artefacts, and altered neurovascular coupling [84]. The ability of AI-based tools to generate artefact-free fMRI maps will improve with the size of data used in the training stages. This will benefit from existing large fMRI datasets and data sharing initiatives. Furthermore, such AI-based tools for artefacts correction in fMRI maps can be integrated with other AI tools that are powering robotic surgery systems, which can ultimately offer an automated robust integrated platform for neurosurgical procedures in patients.

**Fig. 3:**
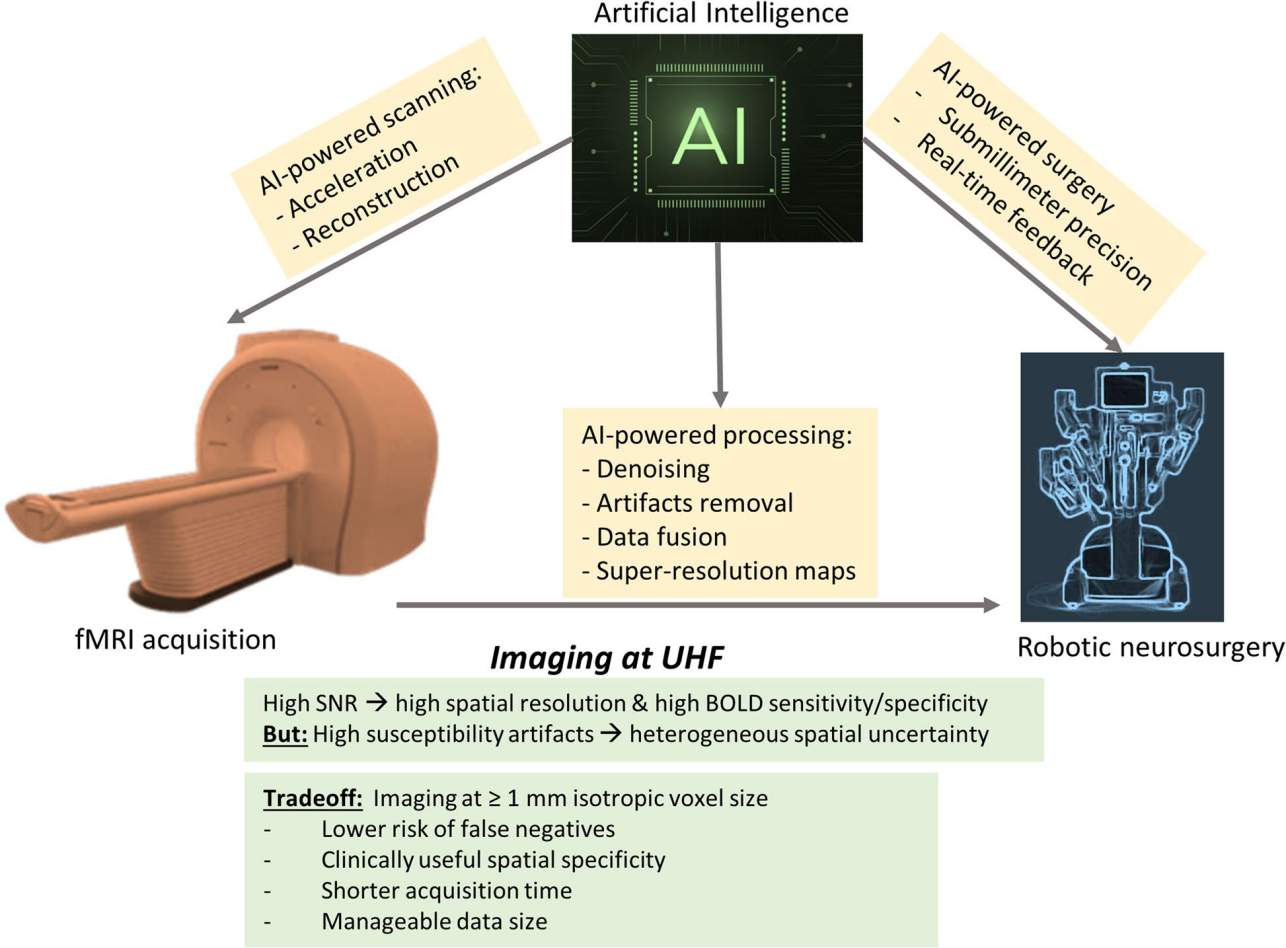
Schematic illustration of the synergy between three advanced technologies for presurgical mapping with fMRI: scanning at UHF, AI, and robotic neurosurgery (yellow boxes). AI can enhance the performance of both the presurgical mapping with fMRI during data acquisition and processing, and robotic surgery for better precision, modelling of brain tissue deformation, and real-time control of surgical tools. [green boxes] main benefits and challenges at UHF, with a trade-off to scan at ≥ 1-mm isotropic instead of aiming at much higher submillimetre spatial resolutions. *AI* Artificial intelligence, *BOLD* Blood oxygenation level dependent, *fMRI* Functional magnetic resonance imaging, *SNR* Signal-to-noise ratio, *UHF* Ultrahigh magnetic fields

In addition to the utility of AI-based tools for artefact removal, recent work also demonstrated the usefulness of AI tools for fMRI maps enhancement, including the possibility to generate super-resolution images [85–87]. A recent work used a deep learning-based super-resolution technique to translate low-resolution fMRI images into high-resolution fMRI images [88, 89]. Specifically, fMRI activations in the motor cortex [88] and the visual system [89] were mapped at much higher resolution after AI transformation than the original images, offering an improved spatial accuracy for the detection of brain activations. This means that generating fMRI maps at submillimetre scale can be achieved from original fMRI data collected at isotropic > 1-mm resolution, hence offering both high BOLD sensitivity at acquisition stage and high spatial resolution at the AI-based data processing stage. AI can also help optimise coregistration or fusion between the generated super-resolution fMRI maps and the collected high-resolution anatomical images for accurate visualization of eloquent cortex. An alternative approach is to display anatomy using a high-resolution EPI image with a strong T1-weighted contrast. For instance, the first collected EPI image of a typical fMRI session, before reaching tissue steady-state magnetization, can display a better contrast between different brain tissue classes. Recent work has also proposed alternative T1-weighted EPI images with excellent contrast and spatial resolution at UHF [90, 91] that can reveal the anatomical location of activated regions with high fidelity. Such AI tools can be made compatible, at high interoperability, with other AI tools that power the robot-assisted surgery system [92] in a way that optimises synergies between fMRI mapping and surgical procedures (Fig. 3). Overall, the implementation of AI to neurosurgery can further augment the capabilities of neurosurgeons and ultimately improve safety and patient outcomes; for review, see [92–94].

## Implications for image-guided robotic-assisted neurosurgery

There is a rich literature about the role of robotic systems for safe and high-precision neurosurgery [95–98]. Robots, usually deployed as robotic arms, are increasingly used in the operating room [99], with image-guided robotic surgeries offering clinically useful accuracy and surgery time [100] as well as high reliability and efficacy [101, 102]. They can eliminate errors [103] and reduce human hand tremors for a higher degree of precision [104]. Despite their limitations with respect to high cost and lack of haptic feedback [105, 106], robotic systems like ROSA, da Vinci, and NeuroArm are routinely used for diverse neurosurgical applications, including in functional neurosurgery, epilepsy surgery, deep brain stimulation, laser ablation of brain tumors, spinal surgery, and stereotactic biopsy [107–109].

In particular, in stereotactic brain tumors resection [110, 111], safety and outcomes for patients have improved thanks to the capability of robots to provide superior spatial resolution, geometric accuracy, and superior dexterity [104, 112]. These capabilities are further enhanced with medical imaging and navigation technologies, thereby yielding robust and safe image-guided robotic neurosurgical procedures [102]. Specifically, such critical imaging information, when offered at high resolution, can help neurosurgeons enter the brain with the robotic arm along the safest angle that would minimise the risk of damage to critical tissue. When those decisions on how to enter and resect the brain are based on presurgical maps, one can envisage the significant benefits that presurgical mapping at UHF might bring with its high resolution and sensitivity, in particular when it is further enhanced with AI [113]. Add to that the possibility to simulate virtual robotic surgeries based on high-resolution presurgical maps, which can then be subsequently executed as pre-planned procedures in the operating room.

Despite the lack of empirical studies on the usefulness of high-resolution presurgical maps at UHF for robotic-assisted neurosurgeries, the gain in resolution and sensitivity at UHF is expected to improve accuracy. We note however that the consistency of robots in achieving high accuracy can vary across robotic systems [114]. Moreover, other advancements are being introduced in robotic-assisted neurosurgery to improve accuracy and safety, including advancements in medical imaging, machine learning, augmented and virtual reality, enhanced interfaces, improved ergonomics, and optimized visualization techniques [98, 99, 115]. Perhaps one important advancement concerns the blending with AI, thereby opening new horizons toward AI-powered autonomous robots in the operating room [106]. For instance, in a recent retrospective appraisal of 700 robot-assisted stereotactic surgeries, the margin errors were on average around 1 mm [116] but were further reduced when robotic surgery was powered by AI [117]. The combination of presurgical mapping at UHF with AI-powered robotic neurosurgery will likely revolutionize neurosurgical procedures for patients with brain tumors or drug-resistant epilepsy. A similar rationale about AI-powered robots was described for endovascular neurosurgery [118]. Such neurosurgical procedures can capitalise on the integration (or fusion) of presurgical fMRI maps with other imaging modalities within surgical navigation systems [119, 120].

## Manageable fMRI data and shorter acquisition times

There are other practical aspects that makes a > 1-mm isotropic spatial resolution a good compromise for fMRI-based presurgical mapping. Going to submillimetre scale at 0.5 mm isotropic for instance will generate massive data that are not always easy to handle in typical clinical settings in terms of data storage and data transfer to surgical navigation systems: acquisition at 0.5-mm spatial resolution generates eight times more data than at 1-mm resolution. For example, a recent high-resolution anatomical image collected at UHF of one subject at 0.1 mm resolution took almost 2 terabytes of raw *k*-space data [121], a storage size that needs to be multiplied by hundreds for raw and processed EPI images. Furthermore, at 1-mm spatial resolution, scanning at UHF can reduce the total acquisition time. This is very useful for task-based fMRI, particularly as many patients find it hard to perform tasks and remain still for prolonged periods [12]. Patients tend to move more than healthy subjects, and many of the tasks used in presurgical planning, such as hand movement or overt speech, might induce head motion [84, 122]. Shorter acquisition times can be achieved by scanning at high temporal resolution (short repetition times), which is already showing improved BOLD sensitivity for presurgical mapping at UHF [123, 124]. Last but not the least, shorter acquisition times will make the presurgical mapping safer for patients by minimizing some of the many side effects that have been frequently reported at UHF [13, 125], including vertigo, dizziness, false feelings of motion, nausea, nystagmus, magnetophosphenes, electrogustatory effects, light flashes, metallic taste in the mouth, too much noise during longer image acquisition, and/or discomfort.

## Conclusions

Current technology at UHF is enabling data collection at high SNR and high spatial resolution [126, 127]. In the next decade, magnetic field strengths are expected to go even higher than 7 T to reach 10 T or even 20 T [128, 129]. This growing interest in UHF is already promoting the development of novel analysis methods to detect atypical brain activations [130]), to generate reliable patient-specific fMRI maps in cases with altered hemodynamic responses [131] or with huge head motion artefacts [132]. Thanks to the increase in BOLD sensitivity at UHF, presurgical mapping with fMRI at 1-mm isotropic resolution offers high BOLD sensitivity, accurate spatial mapping, and manageable data size. Generating artefact-free brain maps is of paramount importance to minimise postsurgical complications [70]. In this context, there is a need for the creation of a task force to develop standardized safe fMRI protocols at UHF, including patient-tolerable fMRI paradigms, low-risk data acquisition protocols, and optimal data processing methods. Validation studies are also warranted [27], in particular for resting-state fMRI protocols. The current growing interest in MRI-compatible robotic systems [133], allowing for instance the deployment of robotic systems inside the bore for a closed-loop surgery architecture, might expand in the next decade to new MRI systems at UHF. Combination will other functional modalities warrants future research [134]. Future work needs to explore novel ways to optimise the synergies between fMRI-based presurgical mapping, robotic neurosurgery, and AI.

## Data Availability

No datasets were used or generated for this study.

## Abbreviations

AI: Artificial intelligence
BOLD: Blood oxygenation level dependent
EPI: Echo-planar imaging
fMRI: Functional magnetic resonance imaging
SNR: Signal-to-noise ratio
UHF: Ultrahigh magnetic fields
